# Epidemic Cholera—Its Pathology and Treatment

**Published:** 1832

**Authors:** 


### Art. VI.—*Epidemic Cholera:—Its Pathology and Treatment.* An Eclectic Review.

Reversing the common order of medical disquisition, we propose to reserve the ætiological history of Epidemic Cholera for our next number, and proceed, at once, to its pathology and therapeutics. In doing this, we shall place before our readers, at the opening of the summer (the season for those varieties of the Cholera which annually prevail in the United States) an abstract of the experience of the profession in Epidemic Cholera; which cannot fail to be useful, in case its remote cause, whatever it may be, should modify or aggravate the endemic, which, as usual, we may expect in June and July.

We shall arrange our selections under the following heads: 1. *Symtomatology.* 2. *Morbid Anatomy.* 3. *Pathology.* 4. *Treatment.* 5. *Prevention.*

1. Symptomatology.—-No experienced physician of this country is ignorant, that Cholera Morbus and Cholera Infantum, frequently prove fatal, within 24 or 48 hours after the attack. Such cases, however, are few in number. If they were greatly multiplied, our summer endemic would, in reality, become identified with the terrific malady which is said, within the last 15 years, to have consigned to the tomb more than fifty millions of the inhabitants of Asia and Europe!

The most extended work on Epidemic Cholera which has fallen into our hands, is by Mr. Charles Searle, a Surgeon in the East India Company's service, and entitled "Cholera—its Nature, Cause and Treatment"—London, 1830. The author while resident in India, suffered a severe attack of the Epidemic, in his own person, and saw much of it in others. To the observations thus made he has added copious extracts from the reports of other British practitioners in that country, so that his book presents a digest of the observations and experience of a majority of those, who have seen more of this pestilential scourge, than all other physicians.

As a general description of the disease, we shall extract from the fourteenth and subsequent pages of Mr. Searle's book, that given by Mr. Orton and avouched by our author:—

"The attack of cholera is usually sudden and violent, but in a great majority of instances, not without some premonitory symptoms —it is frequently preceded by a simple diarrhœa, continuing several days, and still more commonly by other slight affections which are more characteristic of the disease; an extraordinary depression of spirits, and general uneasiness come on, attended by tremor, and sense of debility; giddiness and head-ache, and occasionally ringing in the ears, are also felt, particularly on rising from the recumbent posture, or making any sudden movement. Pains, resembling those which attend the accession of fever, are freqently felt in the limbs; the bowels are griped occasionally, and natural loose stools occur; and nausea comes on. The circulation, and temperature of the body are variously disturbed, but most commonly, the pulse is accelerated and weakened; the skin is moist, and colder than usual, to the hand of another.—These symptoms, or some of them, not unfrequently continue many hours, or even a day or two, without proceeding much farther, or exciting much attention.

In general, however, the severer affections quickly set in, or as he at the commencement observes, the attack is sudden and violent. Acute griping is felt; the stools become frequent and watery, and soon change to the conjee-water appearance; vomiting comes on, of the same mucous fluid appearance; copious sweat breaks out, and anxiety and debility rapidly increase; and the countenance assumes a peculiar shrunk ghastly appearance. It is usually during a fit of vomiting, that spasms of the muscles are first felt, they are almost invariably of the tonic kind, recurring in paroxysms, of the duration of a minute or two, and a tintervals of a few minutes; and attended with excruciating pain; they affect occasionally and in turns, the whole of the muscles of voluntary motion, but particularly those of the legs and feet: the abdominal muscles, and those of the chest are frequently affected, but not particularly so: great debility of the circulating system, very constantly attends the accession of spasms, or severe vomiting: I have frequently found, the pulse sink at once, on the first appearance of spasm in the muscles, from the natural state so as scarcely to be felt, or even disappear entirely from the wrist in an instant: and in such subjects as are not affected with spasms, this fatal failure of the circulation not unfrequently appears, with the first paroxysm of vomiting. The frequency of the pulse is very variable, in many instances it is rapid from the commencement, in others its frequency is little changed in the early stage; but in all, it becomes extremely quick as the disease advances.—The respiration from the first severe accession, is observed to be hurried and oppressed, and is frequently complained of. As the disease increases in violence, the colour of the whole surface changes to a livid hue, particularly round the eyes and at the extremities. The surface is bathed in cold sweat; the hands and feet, and afterwards the whole body, rapidly grow cold.

A small number only of the symptoms of this disease, have yet been described, and it is necessary to return to the early stage, when the spasms, and other severe affections first set in; from this period, an extreme thirst invariably attends; and notwithstanding the coldness of the body, there is an ardent longing for great quantities of cold water; an acute pain is felt in the situation of the stomach, which has been generally and distinctly observed to be increased on pressure, and sometimes on inspiration. A sense of heat is likewise felt in the same situation, and frequently extending over the whole abdomen; which has been compared to the effects of burning embers, or a blister in the stomach. Great oppression, and sense of anxiety are also preferred to the præcordia; the secretion of urine, and of bile under the existence of the severer symptoms, is completely suppressed: the tongue is natural at first, but in the course of the disease, it becomes furred, and deficient of moisture; and dryness of the mouth and throat, is very generally complained of: the hands are sodden with cold sweats, shrivelled and shrunk, like those of a washerwoman after a day's labor, and frequently of a very dark blue colour: although the temperature of the body is greatly diminished, the patient is not sensible of it, on the contrary, he frequently complains of heat, and is incessantly throwing off the bed clothes; these movements are, however, more occasioned by an extreme restlessness and anxiety, which prevent his remaining an instant in the same position; it appears to be attended with such an extreme degree of suffering, as human nature cannot long support; and accordingly, we seldom find it of long duration: it is gradually relieved, or removed by stupor, and though these symptoms are of so opposite a nature, they are frequently present, in a very considerable degree at the same time: the patient feeling a constant inclination to dose, and waking every instant, tossing and groaning in a dreadful state of anxiety.

On the accession of the spasms, or, in those subjects in whom they do not appear, of the vomiting, the other symptoms above described, usually either make their appearance, or undergo a marked exacerbation. The disease at that period, seems to put on its most acute form, and the great struggle takes place.—It is quickly decided—after an uncertain continuance of this state, to which I cannot with truth, apply a term of greater precision, than a few hours, a remarkable change takes place, which might lead a practitioner unacquainted with the disease, to congratulate himself on having arrested the worst symptoms, and to form a favourable prognosis, even when death is approaching with rapid strides: the spasms, the vomiting, and the purging cease, usually about the same time. Whatever is taken into the stomach is retained, even though in large quantity; and clysters, not rejected as formerly. About this time likewise, the state of dozing and stupor which is described as above, appears, and considerably resembles natural sleep. But any favorable inference which may be drawn from these appearances, is quickly contradicted, by the continued weakness of the circulation, the coldness and livid colour of the surface, and the cadaverous expression of the countenance.

The powers of life continue rapidly to fail—the pulse becomes quite lost at the wrist, and even at the humerus; the pulsation of the heart itself is felt extremely feeble; the eye is sunk back and fixed in its socket, the cornea becomes dull and glassy, or is covered with a tenacious film. From the shrinking and wasting of the features, the eyelids are rendered incapable of performing their office, and in the attempts to sleep, the eye is half open: deafness, preceded or accompanied by tinnitus aurium, is very common in this stage: false vision, blindness, and dilated pupil, are equally so; speech becomes difficult, and the voice grows hoarse, hollow, and scarcely intelligible, or entirely lost. The breath has been observed to be cold, "as if it came out of a lump of clay." The internal senses are occasionally observed to survive the other faculties; but more frequently they are absorbed by stupor. Respiration is short and laborious, and frequently stertor supervenes—after a further continuance of some hours, with various degrees of the preceding symptoms, death closes the scene; usually with little suffering.

If the termination is different, a favorable crisis occurs, which is almost invariably marked by sleep of unusual soundness; attended by warm perspiration, and light and natural respiration—the favorable change takes place, at all periods of the disease; but most frequently, before that morbid quiescence with its attendant symptoms, which have been noticed; for this state, is very commonly followed by death; on waking, the patient feels himself thoroughly relieved, and expresses his satisfaction in the strongest terms—at the same time an evacuation of bilious fœces, and urine generally takes place; and from this period, a considerable purging of black, green, or yellowish feculent matter arises. At this time also, other signs of increased action manifest themselves: the pulse rises above the natural standard, both in frequency and volume; the skin grows rather hot, though moist, and frequently there is a copious perspiration.

The Bengal Medical Board have given a luminous description of this stage of excitement or reaction, which in a greater or less degree, appears always to follow the favorable crisis: they have observed it rising to a great height, assuming all the characters of the idiopathic bilious fevers of the country, and occasionally becoming fatal. This, however, does not appear to have been commonly the case on the other establishments—a slight and salutary degree of reaction only, has usually followed, which has quickly subsided; a rapid convalescence has generally ensued, and the strength and looks have been regained, almost as quickly as they were lost.

A still more rapid and violent description of the disease has frequently been observed, particularly in natives:—in a state of health when the patient is going about his usual occupations, the sensorium is in an instant invaded, by violent vertigo and ringing in the ears, together with deafness, and dimness of sight. The contents of the bowels are discharged, much diluted, at one very large evacuation; after which, the white stools characteristic of the disease immediately appear, vomiting and other violent symptoms at the same time set in, and the most extreme debility, and even death is produced in the course of half an hour. The first appearance of the disease in His Majesty's 34th Regiment, was marked by a single case, which was fatal. On the two following days no attack was observed; and the alarm which was excited, had began to subside: but on the day succeeding, thirty-seven men were carried in general nearly lifeless to hospital, between morning and midnight: in that short space fifteen of them died. It would appear, that when the spasms are very violent and general, and the retching extremely distressing, that purging is little remarkable, and frequently altogether absent.

Another very striking set of features of this proteiform disease still remains to be delineated; and although I have deferred, for the sake of perspicuity, the mention of them until now, they are amongst the most important, and most constant in occurrence.—The burning pain in the stomach is increased on pressure, and the common circumstance of the ingesta being instantly rejected, are indicative of inflammation of that organ; and the appearances on dissection fully prove, that this affection takes place, perhaps in every instance; for, whenever the disease has been of sufficiently long duration, to allow time for appearances of inflammation to be formed, they are invariably found, not only in the stomach, but in the intestines. In many instances, we have the most convincing proofs during life, of the existence of inflammatory action, in different parts of the frame: if the extreme depression of all the powers of life already described, and particularly if the state of torpor, which follows the morbid cessation of vomiting, purging, and spasm, is not quickly concluded by death, or natural sleep, the temperature from being far below the natural standard, rises above it. If there is any vigor of the circulation left, the heat extends over the whole surface, and the moisture disappears; but if the powers of life are unequal to this, the trunk only becomes hot, the extremities continuing quite cold, and moist. Under these circumstances, the pulse is also extremely quick, 140 and upwards, sharp, and occasionally possessing a peculiar irritated thrill, which is strongly expressive of inflammation of vital parts. The tongue grows furred and dry, and in combination with these symptoms, many local appearances occur, leading to the same conclusion.—In some cases the vomiting returns, and continues very frequent, the stomach rejecting every thing which is taken in, for several days: in others, there is fixed pain, and soreness all over the abdomen; and if the inflammation attacks chiefly the brain, there is occasionally muttering delirium, but more frequently, coma, with deep and stertorous breathing, and suffusion of the conjunctiva, which quickly terminate in death. In cases which have lingered on for some time, in any of these states, and even when the topical symptoms are scarcely at all evident, the appearances of inflammation on dissection are strongly marked, and quite sufficient to account for death.

This inflammatory stage frequently lasts several, even many days.

If the depression of the vis vitæ is extreme, the secretions of bile and urine, and perhaps nearly all the secretions, continue suppressed throughout. Under other circumstances, I have observed, small quantities of high colored urine frequently passed; and black or green stools occur, without a favorable termination. On the commencement of this stage, the pulse usually becomes rather more distinct; but it is more from increased sharpness, than fullness: and we are presented with the remarkable phenomena, of acute inflammation, and extreme debility prevailing at the same time, and indicating opposite modes of treatment.

On the subject of Inflammatory Symptoms, Mr. Searle makes the following additional remarks:—

My own experience has also been very conclusive with regard to the sthenic form of the disease.—I have found a very considerable number of cases, exhibiting singly or in partial combination, every possible degree, and almost every kind of increased action.—Spasms, such as not to fall short of the highest degree of tetanus; retching, and spasms of the intestines equalling colica pictonum; very full, hard, and quick pulse; hot skin and flushed surface;—evacuations of bile, both by vomiting and stool, from the commencement of the attack.—And finally, I have seen some of these cases passing into the low form of the disease; the circulation passing suddenly, from extreme strength to extreme weakness; the skin from being hot and dry, becoming deadly cold, and bathed in sweat; and the increased flow of bile, succeeded by white stools, and vomiting, clearly indicating the total suppression of that secretion.—The inference from these facts is plain; however opposite these two forms of the disease may appear, there is no essential or generic difference between them.

The manner in which Mr. S. presents the following remarks, leaves it uncertain whether they are from his own or the pen of Mr. Orton:—

As it has been said, that the preponderance of increased action marks a milder form of the disease, and that the European constitution more frequently exhibits that phenomenon, than that of the native, it follows, that the disease should be less fatal in the former, than in the latter class of persons, which experience proves to be the case.

In fact if would appear, that increased action in general, whether arising from a smaller degree of morbific cause, or greater vigour in the system, is equally indicative of less disease. It is evident, that the quantum of disease in any case, must be, not only in proportion to the quantity of external cause, but in the inverse proportion to the power of resistance against it, inherent in the frame. The most vigorous and healthy, are incontestably the least subject to this epidemic, or when attacked, to its more dangerous forms; and it is probable on the foregoing principles, that these previous states of the system, by affording resistance to the morbific influence, reduce the disease to its milder form,] and thus chiefly give rise to the same phenomena, as those which attend the diminution of the great cause of the epidemic.

It appears, therefore, that the great and ordinary diversities in the phenomena of cholera, which arise from unusually increased or diminished action, are principally referrible to different degrees of disease."

That our readers may compare the phenomena of this disease, in climates so opposite, as those of Bengal and Russia, we shall present them with its symptomatology in the latter country, as given by medical gentlemen sent thither by the French, Prussian and English governments; for the two first of which we are indebted to the documents recently published by Congress; for the last, to the distinguished Med. Chir. Rev. for January 1832.

Dr. Perron the French agent writes as follows:—

*Ordinary course of the Asiatic Cholera.*

We may admit of three stages in this plague. The first is that of its inferior degree, of its imperfect development, or only of the *precursors.* The second embraces its ordinary development. And the third comprehends cases, in which the most alarming symptoms of the former stage acquire the greatest degree of intensity. The first period is, unfortunately, too often neglected, at the great peril of the patient, because the symptoms are not then sufficiently alarming to give uneasiness; they are not even considered as belonging to cholera, but as the consequence of a simple gastric affection. The symptoms belonging to this first stage, are a sensation of uneasiness, either with, or without pain in the pit of the stomach, the want or loss of appetite, a great heaviness in the lower part of the abdomen, with a disposition to diarrhea or a diarrhea partly developed and which may precede the attack for some time; afterwards some nausea, and even sometimes slight vomiting, and weakness in the limbs, head-ache, alternate shivering, and slight heat. These symptoms are not always united; sometimes one, and sometimes the other, predominates. During the epidemic, they are found more or less in every body; they last several days, disappear and return, and often have no other effect. They must then be regarded as an imperfect development of the disease.

The second stage is that in which the malady presents itself in its most striking characteristics. Here too, the symptoms are not always the same in all individuals; but the following are generally observed: frequent and weakening evacuations, both by vomiting and purging of aqueous liquids, greyish, like rice water, commonly untinged with bile, inodorous, and connected with some particles of mucus. At the same time, respiration becomes more and more difficult; and this state is followed by excrutiating pain, stifling and constriction in the region of the heart, interrupted by sighs. In the lower part of the abdomen, the patient experiences, alternately, pains accompanied with heat; the disposition to vomit and purge augments, whilst the urine ceases entirely. Thirst becomes unquenchable, and with it, a strongly marked desire of taking cold drink, and chiefly water, to calm the burning sensation in the pit of the stomach. Restlessness augments to such a degree, that the patient hardly remains more than a single moment in the same position. The tongue becomes flabby, large, bluish, and even blue; afterwards it grows cold as well as the breath. The extremities likewise soon grow cold, bluish, then blue, and are wrinkled as if they had been for a long time in warm water. The patient experiences at first pains and heart burnings, to which succeed very severe cramps, chiefly in the calf of the legs, in the joints of the toes and fingers: they are afterwards felt in the thighs; in the abdomen, and in the inferior part of the stomach. The pulse lowers, and becomes almost imperceptible; the eyes are blood-shot, dim, hollow, and surrounded by a dark circle. The face, which is also blue, contracts, and expresses both the increase of weakness and of the collapse, as well as the greatest sadness, and the anguish of an imminent death. The blood drawn from the veins is black, and so thick, that it runs with great difficulty, and is cold to the touch.

The third stage is that of a new degree of intensity in all or in a part of the symptoms detailed above, or there is a deceitful appearance of amelioration: that is, the evacuations and the cramps cease, and the patient no longer feels any pain, but the skin remains as cold as marble, the respiration becomes more and more weak, and there remains no trace of pulse.

The indications of amendment, consist in the diminishing of the cold of the body and extremities, in the reappearance of the pulse, however feeble at first; in the gradually ceasing of the alvine evacuations and vomiting, and chiefly in the reappearance of the urine.

The patient recovers sometimes very fast, that is in the course of a few days; but very often too, several weeks are necessary to his entire restoration. In short, it is also in that stage that the disease, by a reaction in the system, changes into a febrile malady, accompanied by a strong affection of the brains, or of the lower part of the abdomen, which under the appearance of a nervous typhus, still presents a great deal of danger.

The progress of the Asiatic cholera is so rapid, that the fate of the patient is commonly decided in a few hours; some die after four, seven, ten, or twelve hours; it is seldom that cholera lasts more than two days.

Dr. Albers, the head of the Prussian Commission, after observing the disease in Moscow, gives the following summary of its symptoms:—

*Symptoms of the Disease.*

General uneasiness; violent head-ache and giddiness; great languor; oppression at the chest; pain at the pit of the stomrch and at the sides; a very weak pulse, and frequent vomitings, first of undigested food, and then of a watery fluid mixed with phlegm; frequent purging; severe pains, which make the patient roll about and scream; cessation, or very scanty secretion of urine; excessive thirst; cramp in the legs beginning at the toes, and by degrees reaching the body; voice feeble and hoarse; the eyes dull and sunk in the head; the features changed, and like those of a corpse; coldness; contraction and blueish tinge of the extremities; a coldness over the whole body; the lips and tongue become blue; a cold and clammy perspiration. The vomiting and purging soon exhaust the strength of the patient. The spasms become greater, attacking successively, the most vital parts. The pulse ceases, the beating of the breast becomes scarcely sensible, and the patient, after having suffered the most horrible martyrdom, dies quietly, having a few minutes ease just before his end. The duration of the malady is. generally speaking, from twenty-four to twenty-eight hours; but sometimes its course is still more rapid, and sometimes slower.

Dr. Hamett, Naval Surgeon, and one of the Commissioners sent by the British government to Russia, gives the subjoined description of the disease as observed at Dantzick, in Prussia:

Passing over subordinate features of the epidemic, I shall limit my descriptions here to three principal forms of it; viz.

1. The rapid and severe cases of fatal cholera.
2. The protracted cases of fatal cholera; and
3. Those less severe, which proved favourable.

1. In most rapid and severe cases of fatal cholera, the patient was suddenly seized with sickness or pain at stomach, occasional pain, or feeling of weight and uneasiness in the hypochondria, the right hypochondrium especially; giddiness, prostration, great thirst and craving for cold drinks, a cold sweat that quickly became colliquative and clammy; at times coldness alone, at others coldness and dampishness of the body, but never with shivering; the pulse was frequent but not hard, and soon became exceedingly reduced; the hands and features somewhat shrunk: the tongue was foul, unnaturally moist, and occasionally tremulous: the voice subdued; the eyes heavy and suffused, and the sight dim. These primary symptoms were in general either accompanied, or immediately followed, by retching and vomiting, and a peculiar watery diarrhœa, which often, however, proved irregular in the order of attack, occasionally even with respect to each other, and oftentimes severe, in hot, close, and electrical weather especially; griping pains in the abdomen; painful contraction of the muscles at the umbilicus; suppression of the secretion of urine, and occasional pain in the region of the bladder. Cramps in general followed the retching and vomiting, and in most instances invaded the calves of the legs at first; in their attacks of other parts of the extremities, they proved irregular, seizing first the fore-arms, calves and fore-arms, hand and fingers, toes and feet, or hands, feet, and calves, in different instances indiscriminately; occasionally they mounted up the thighs, but seldom attacked the trunk. Men rarely escaped them, women frequently, and children generally.

The vomited matter in general consisted of undigested food at first, sometimes partially tinged with yellowish green; of fluid ingesta, also occasionally imbued with greenish coloured matter, and partly of slime and mucus. Often, however, it consisted of undigested food, or of fluid ingesta alone, without being in any wise so imbued. In the retching and vomiting which followed, the fluids taken continued to be rejected with a little greenish coloured matter, with or without more slime or mucus. The dejections, were always watery; sometimes as if coloured with feculent matter; in general they were either colourless, somewhat like whey or had the appearance of rice-water, barley-water, occasionally somewhat dirty, or with an avanaceous sediment, after being shaken in water.

After this first advance of the disease, the following symptoms rapidly supervened; viz. increasing oppression at the heart, and short hurried and laborious breathing, ending in complete oppression and weight at the *præcordia;* tossing of the head about; anxious restlessness depicted, often with terror, in the countenance, which in general was of a dark brown, wan, or leaden hue, according to the complexion; insatiable thirst, with incessant craving for cold drinks, and the voice raucous and depressed. The retching and vomiting, and diarrhœa, with occasional *tormina* and cramps, at first only intermitting at short intervals, subsided either abruptly, or gradually as vital exhaustion advanced; the pulse at the wrist, if not extinct—which it was in most rapid and severe instances—was accelerated to the utmost in frequency, and barely felt; the surface of the body quite cold, damp, and clammy, and the feet and insteps marked with bluish streaks and patches; the tongue cool or cold, and in some instances livid at the tip and edges; breath cool or cold; lips blue; nose sometimes bluish; voice below the breath, or gone; cheeks and eyes now quite sunk; pupils at times partly or completely dilated; eyelids half-closed, and encircled with livid rings; the parts of the conjunctivæ exposed being much the same in appearance as after death. Amid this complicated suffering, the patient was not insensible until just before dissolution, which ensued after some faint convulsive sobs, generally within from eighteen to seventy-two, and occasionally within from eight to eighteen hours after the first attack.

2. In the protracted cases of fatal cholera, which have been few in number compared to the rapid cases, the following febrile symptoms have been observed more or less, in different patients after the indefinite period of the first stage: marked congestion, with pain in the head, deafness, humming noise in the ears, heavy stupor, continual drowsiness, partial ravings; a dark flushed, brownish yellow, squalid or cadaverous countenance; a dark brownish clammy or furred tongue; dark sordes about the teeth and lips; eyes heavy and suffused, or dry and parched, often with eventual dilatation of one or both pupils; a hot or cold clammy skin; pulse frequent, with febrile action, or very small; with pain or soreness of the abdomen, increased on pressure—and occasional tenesmus. With these symptoms, the excretions, as may be readily conceived, were scanty and vitiated. The stools dark, dark-green,—very fetid, and the urine in general dark coloured. Delirium generally took place in these before death, and they died within from three or four to five or seven days, or later, after the first attack; more generally on the fifth.

These modifications of particular symptoms, bordering on each other, and referring to individual parts, depend, I need scarcely add, not only on differences in constitution, but, in a certain degree, on the mode of treatment at the commencement, and even on the state of the locality in which the patients happened to be placed.

3. In the cases less severe,—and as I have observed,—of less unhealthy persons, in whom the natural powers* of the constitution were calculated to withstand the effects of the shock on the system, giddiness, retching and vomiting, watery diarrhœa, occasionai griping pains in the abdomen, cramps, occasional painful contraction of the muscles at the umbilicus, thirst, and suppression of the secretion of urine, took place, and proved occasionally severe; but the congestion in the head, and oppression in the chest were certainly less marked; the pulse, although barely felt, was rarely entirely suppressed; coldness of the body, the cold clammy sweat, and other bad symptoms were not marked in any great degree. The leading symptoms gradually, or abruptly disappeared; and more or less of febrile re-action ensued, generally within from eighteen to twenty-four or thirty-six hours, or more, after the commencement of the disease, about which time hiccups, always a favourable sign, were occasionally noticed, and not before. The exact period of the return of the urine was not certain, being sometimes before, at others after the first appearance of re-action: it was dark or high-coloured, voided in small quantittes with occasional difficulty, and frequently attended with some pain in the region of the bladder. The return of urine, though an important symptom, was not always decisive of a favourable result; on the contrary, hiccup, which however was not always observed, almost invariably indicated recovery. The dejections immediately after the commencement of re-action were fluid, scanty, and dark-coloured, as if imbued with blackish feculent matter; but they very soon became successively brownish, and naturally bilious and feculent. Indeed, in the majority of cases of this description, the secretions and excretions soon got into play, and restoration was more or less rapid.

Partial stupor, with little or no delirium, more commonly occurred in children, and spare aged persons free from previous organic, or

*This is certainly true; and yet a woman, named Eliza Brandt, thirty-six years of age, affected with tubercles and vomicæ, had, in July last, this severe form of cholera, which soon gave way to all the hectic symptoms of her complaint.

general complaint, and gave grounds for a favourabte prognosis: they seemed tranquil, and as if naturally asleep. They were in general affected with *œdema*, in the feet, and more or less in the legs after convalescence had commenced. *Œdema* also occurred in others after the disease, but not generally. In pregnancy, abortion invariably took place, and was always a critical symptom, death or a favourable change soon following.

From the description above given of the rapid and severe cases of fatal cholera in Danzig, its similarity to the Indian cholera appears manifest; and from the descriptions of fevers supervening after the first stage, as given in the second and third forms, its deviation from the Indian epidemic, in which those fevers do not generally supervene, also appears evident. The greater severity in general, which has been found of the vomiting, diarrhœa, griping pains in the bowels, and painful contraction of the muscles at the umbilicus, in the epidemic in India, compared to that in Danzig, is easily explained by the well-known influence of the climate in India on the whole system, and digestive canal in particular.—*Med. Chir. Rev. Jan.* 1822.

II. Morbid Anatomy. Mr. Orton, as quoted by Mr. Searle give the following account of the morbid appearances presented after death, in India:

The morbid apearances which I have observed on dissection, in a great number of instances in this disease, have been pretty uniformly as follows:—

A dark blue, or livid color of the surface of the body, existing in various degrees, in different parts of different subjects, but most remarkable at the extremities. It appeared to prevail most in the more robust and sanguineous subjects, and in the more rapid cases. Blood, taken from both the venous and arterial systems, was found to be of a very unusually dark, and purplish color. The internal organs in general, were found much gorged with blood—this was particularly remarked in the veins of the mesentery and stomach, and in the lungs.

Many extensive patches of a crimson color, on the stomach, usually confined in a great measure, to its inner coats; similar appearances in the intestines, most remarkable in their inner surface, but appearing externally in a greater degree than in the stomach—they were much more evident in the small,, than in the large intestines.

The stomach, containing the ingesta which had been given, for some hours before death in considerable quantity, and little altered, either in appearance or smell: calomel was frequently found at the bottom of the fluid contents, and adhering at various places to the mucous coat. The intestines were in a great measure empty; their contents were usually free from the slightest tinge of bile, and consisted chiefly, of a dirty whitish mucus, resembling conjee or thick barley water, slightly turbid with milk: large portions of them were frequently found contracted, so as with difficulty to admit the finger into their cavities, particularly of the large intestines. The gall bladder contained the natural quantity of bile, of no very remarkable appearance. The gall ducts were found pervious; the bile flowing readily into the duodenum on pressure being made on the gall bladder.

The urinary bladder was almost invariably found contracted to the size of a hen's egg; without containing a drop of urine.

The veins of the brain were much distended with black blood: the minute arteries of the dura mater, and membranes of the brain were frequentty found injected. I am informed that actual extravasation of blood, in considerable quantities, has in some instances, been found on the surface of the brain.

Let us compare these with the appearances, on dissection, in the North of Europe. Mr. Hamett examined 21 subjects, of various ages, some of which died from sudden and violent attacks—others from protracted illness, and has reported the morbid anatomy as follows:—

Many of the characteristic appearances after death will depend, in a great measure, upon the number of hours elapsed before the body is opened; the later the examination, the less truly characteristic, so far, are the appearances. Those I have examined were in general opened within, or about, twelve hours after death. Bodies at this season ought, however, to be examined as soon as possible, and always within at least six hours, if it can be done with propriety.

Of all the morbid effects in appearance, which I have observed after death in the bodies of persons who died of cholera in Danzig, the most characteristic, perhaps, has been the great congestion of blood in the sinus venosus and right auricle of the heart, and in the veins throughout the whole body; the next is the invariable contraction of the bladder; and another, which, although not apparently constant after death from this disease, is seldom or never to be met with after death from others—namely, slight spasmodic contractions, or movements, if they may be so called, in the muscular fibres here and there in the body, and more especially in the face and extremities, not only immediately, but some time after dissolution. These resemble Galvanic effects produced in the body after death.

The veins and right auricle in particular of the heart, were full of black blood; some was always found in the left auricle; while very soft imperfectly coagulated lumps were found either within the right ventricle, or within the aorta, either immediately at its commencement, or down below its curvature. These lumps were invariably as black as the blood found in the veins and right auricle; the thoracic aorta uniformly contained some black blood, but was never full, like the veins; the abdominal aorta also contained a little, but very little; the right ventricle had always a small quantity of black blood, the left ventricle a very little. The pericardium seemed more or less flaccid and very often contained a quantity of dark brown serous fluid. The parietes of the heart in general seemed soft, and I fancied, in a few instances, that those of the left auricle seemed thickened; this, however, remains to be confirmed or refuted by subsequent examinations. I occasionally observed morbid blackish, or bluish, and, in one instance, whitish spots on the external surface of the heart.—The lungs were in general much more bluishly speckled than in most other cases,—almost always collapsed, but dense from black blood—not as in hepatization of the lungs—frothy, black blood freely oosing from incisions made into them. The pleura, in its reflections throughout, from the anterior to the posterior mediastinum, and over the upper surface of the diaphragm, seemed in general of a dark dull red. The trachea, bronchia, and larynx contained a little frothy mucus, and were otherwise wet with a compound of serous and clammy fluid; but the internal mucous surface exhibited no vascular appearance. In general there was a considerable quantity of clammy, serous fluid found effused in the chest; all was wet, exceedingly soft, and clammy, more so than I have been used to see after death from other diseases. The vena azygos was invariably full of black blood. The thoracic duct was in general empty, and seemed natural.

On detaching the calvaria from the dura mater, the latter was, in most instances, spotted all over with the black blood that instantly issued from the torn vessels, especially along the lines of the sutures, where they are most numerous, in the younger subjects particularly. The external surface was mostly of a dark bluish colour, and dry, but clammy feel. The internal surface of the dura mater, and its processes, or continuations, were not marked by any peculiarity, except, perhaps, in the appearances being more opaque, and feeling more clammy than usual. The tunica arachnoidea wrs in general of a wheyey, glossy colour, and somewhat clammy to the touch. Between this membrane and the pia mater, and more especially in the lower part of the cerebellum, there was occasional effusion or filtration of serous fluid; and in all instances there was considerable effusion of this fluid between the pia mater and the cerebrum and cerebellum both; in most instances it was found in the ventricles, in the fossulæ at the basis of the cranium; and, indeed, wherever this effusion between the tunica arachnoidea and pia mater in parts of the cerebellum, and the pia mater and the brain itself at large, was observed, it was also invariably observed in the same relative situations in the spinal marrow of those bodies in which the spine was examined—which were fifteen in number. In other instances too, where there was effusion in the brain, we had only to elevate the pelvis and loins in order to see serous fluid issue forth from the spine through the occipital foramen. There was always a considerable quantity of thin black blood in the sinuses, in the inferior more so particularly. In all cases the congestion of black blood in the veins of the pia mater, in the venæ Galeni, and choroid plexuses, was great, accompanied with varicose dilatation of these vessels; and likewise the same relative congestion of black blood in the veins of the pia mater, in the spine, especially in the posterior parts of it, where the vessels being larger and more numerous, varicose dilatation was more conspicuous. The medullary substance of the brain seemed in some instances much softer than usual, but it might have been owing, in part, to the interval elapsed during hot weather between death and the time of examination. In some instances black spots were visible on incisions into the brain; at times, too, the cineritious and medullary substance both seemed relatively altered in appearance as well as consistence.—The state of the spinal marrow corresponded in all cases exactly with that of the brain.

After what has been said and implied on the venous congestion in the brain, spinal marrow, and thorax, it will be readily conceived in the abdomen, in which the large as well as small vessels are still more numerous and varied. The vena cava abdominalis and vena portæ, with the splenic and superior mesenteric trunks, and, in short, all their large tributary branches, invariably contained a considerable quantity of black blood: they seemed at times as if full of it, while the mesenteric veins always exhibited a characteristic black or bluish arborescent appearance throughout. The gall-bladder was not only of a deep green externally, but, in some instances, from a deep green to a bottle-green, and occasionally tinged here and there with yellow; and was in general distended, and full, or nearly full of fluid, generally black, and sometimes as if a little of yellow or brownish yellow bile had been mixed up in it. The internal or villous coat of the gall-bladder was in general between a dirty yellow-brown and brownish yellow—in a few instances it was a natural bilious yellow. The liver was invariably in a state of *engorgement* from the black blood, which, in all states of it, freely oozed out from the hepatic veins in particular on incisions into its substance: it was in general discolored, even after sponging the membrane covering it, and I think most in the younger subjects, and those who had not suffered from previous affection of it. The spleen was also in a state of *engorgement*, and of a black purple colour—and this independently of any alteration in its structure as referrible to other morbid states. The kidneys, notwithstanding the suppressed secretion of urine, did not exhibit any peculiar change in general, further than that of venous congestion. The same was observed in the pancreas. It is not easy to say whether the ductus communis choledochus, and immediate biliary vessels were in general contracted or not: sometimes I found greenish or vitiated bile at the opening of it into the duodenum, and sometimes I did not. I often found, in protracted cases particularly, the external parts of the duodenum and colon in contact with the gall-bladder, or near it, completely discolored with yellow bile.—With respect to the stomach and intestines generally, I cannot say that I observed any effects of the disease beyond what is referrible to congestion of blood in the veins, and what might be attributed to the sedative nature of disease. The mucous coat of the stomach, in particular, and parts of the colon, seemed, in some instances, soft, as if half macerated, indeed, the intestines generally seemed soft, as if the internal mucous and villous coat could be separated from the muscular coat. The small intestines, I mean the jejunum and ileum chiefly, were more commonly of a dark dull red, or rather of a dark dull slate colour, on their external peritoneal coat, without any positive vascular appearance; sometimes of a pale slate colour, with vascular injection, or vascular congestion more marked; while, on the internal surface, they did not exhibit the same colour generally—still, in some instances, there was in some parts a modified appearance of it; while in various parts in others there was a vascular appearance of the internal mucous and villous coat, though by no means corresponding to that externally. Besides the pale slate or leaden colour, and the dark red slate colour, I have observed a vascular dark red also—facts which will account for that tenderness or pain on pressure of the abdomen, so marked in cholera, especially in protracted fatal cases. In one instance of a young woman, who had died of true and rapid cholera, the general external appearance of the small intestines was of a pale or light rosaceous hue, while that of the colon was quite pale. The mucous membrane throughout the whole canal was whitish, and as if half macerated. Whether the brown patches, which are at times observed here and there on the internal surface of the stomach and intestines, are effects of the disease, or of previous chronic inflamation, is in some instances not easy to determine. The stomach and intestines, as might be expected, mechanically retained the last fluid ingesta; for latterly, what came away, did so involuntarily. There were the remains of former mucus, more or less, throughout the whole digestive canal; and in true, rapid, and fatal cholera, little or no remains of feculent matter, except in its usual receptacles, namely, the commencement of the colon, the cæcum, caput, in the transverse arch occasionally over across it, and in the sigmoid flexure, in which, in some instances, scanty portions of it were found. The mucous follicles in the internal membrane of the colon at its commencement, and Peyer's glands in the end of the ileum, were occasionally found in large compact patches, more or less continuous, distinct, elevated and somewhat indurated. Brunner's glands, as they are called, were not so observed in the duodenum. The colon externally, as well as the duodenum, particularly at its upper curvative, was discolored at the upper part of the ascending portion, and beyond in the greater part of the transverse arch, but in the other parts it was of a pale or pale lead colour. The peritoneum, in all its detached reflections, was more or less opaque, having lost its shining, glossy colour more than in most other congestive and sedative diseases of the system attended with fever, more even than in the compounds of remittent and intermittent fevers in tropical climates, with marsh miasmata, in which venous congestion is so very notorious. In protracted fatal cases I occasionally observed chronic discoloration here and there on the internal surface of the stomach and intestines—in some instances of a dark brown, in others of a dark brown red, without being exactly vascular in appearance: at times vascular spots and patches were observed in some parts of the intestines, and the dark brown, and dark brown red in others; they were generally in the colon, the commencement above and below particularly, in the transvers arch, and sigmoid flexure. I observed parts of the colon in a gangrenous state, and chronic inflamation of the whole of the ileum, in one subject. In several instances the lumbricoid ascarides were found in the intestines.—In some instances the commencement of the thoracic duct or receptaculum chyli seemed quite close and contracted.—The invariable close contraction of the bladder, I have not omitted to mention; it was mostly lined with a little whitish mucous.—*Med. Chir. Rev. Jan.* 1832.

As some patients expire in a state of asphyxia or collapse, within a few hours after the attack, while others live for several days, and die from inflammation; it need not surprise us, that a summary of the morbid appearances shonld present much diversity.

III. Pathology.—Whatever may be the cause of Cholera, its fatal energy must be admitted. The suddenness of the attack; the extreme prostration of the vital forces; the universal overthrow of the functions; the speedy termination in death; and the accumulation of blood in the great central organs, as found after that event, manifest the impress of a most desolating poison. On what part of the living body this agent makes its onset, remains to be discovered. 1. Like electricity it may penetrate our organs, and by its actual presence exert a deleterious influence. 2. It may stamp itself on the dermoid or mucous surfaces, or both, and communicate its baleful influence to the internal parts, through the medium of the nervous system. 3. It may be absorbed through the lungs into the blood, and circulate as a virus; or changing the composition of that fluid, render *it* a poison to the organs which it should sustain. In whichever of these modes it makes its invasion, no part of the body can be said to escape the consequences of its mortal impress; for the symptoms denote that every organ is scathed, though not perhaps in an equal degree.

In reference to our vital properties, is it a stimulant, a sedative, or an irritant? The phenomena which follow its application, may answer this question. They are evidently not those of increased excitement; but the reverse. The activity of the various organs is reduced, and at the same time perverted. The noxious agent is obviously both sedative and irritating. The great function of innervation is almost suspended; and the remnant of sensibility, both animal and organic, altered; the equilibrium of the circulation, as an inevitable consequence, is broken up;—the enfeebled heart can no longer propel the blood to the more distant viscera, the muscles and the skin;—the vessels equally fail in their co-operation, and the vital fluid undecarbonized and impure, stagnates in the great vessels, and the portal and pulmonary circles, in the vena azygos, the spinal marrow, and the brain. Thus the universal and governing functions of innervation and circulation, are deeply smitten and deranged; the læsions they have suffered, reciprocally promote each other; and as every other function is subordinate to these, so every other is of course reduced and perverted. That of the lungs is arrested till the little blood which the organ transmits, is sometimes found of a black color in the aortic ramifications during life; the secretion of bile is nearly, often quite, suspended; the bladder is contracted from deficiency of urine; the skin is dry, or bathed in the kind of exhalation which, in most fatal diseases, precedes death; the healthy mucus of the gastro-enteritic membrane, is replaced by a sero-mucous exhalation resembling barley-water; the calorific function is reduced; to the point of actual suspension in the limbs, which during life sink to the temperature of the surrounding bodies; finally, the contractions of many parts of the muscular system—those of respiration, of the extremities, and of the alimentary tube, become morbidly excited, as in sycope from loss of blood, or hyper-emesis from tartrate of antimony. The various excreta of the blood, including two-thirds of the carbon exhaled in respiration, being retained, contribute to the impurity of that fluid; so that the central organs are not merely engorged, but engorged with a vitiated blood. This contributes to sink still lower their vital powers, already reduced by the direct action of the remote cause, and the patient frequently dies in as short a time, as if a solution of arsenic or antimony had been injected into his veins.

If he survive the stage of prostration, a partial and irregular reaction commences. Inflammations in various parts—chiefly the membranes of the brain and alimentary canal, are set up; while other parts remain engorged with black blood, and inactive. A hot stage ensues, and the symptoms of a typhus gravior are developed. If the patient were previously vigorous, and of a sanguine temperament, the inflammatory symptoms may even run high. Thus the succession of morbid actions is that of malignant fever; and a paroxysm of one of the congestive intermittents, which occasionally scourge certain districts in the western country, is the true prototype of Epidemic Cholera. The chief difference between the two maladies consists—first, in the tendency of the former, to repeat itself in paróxysms; and secondly, in the vomiting and diarrhœa; accompanying the latter; which, however, are not always present, and to a greater or less degree often show themselves in malignant intermittents.

IV. Treatment.—Much has been said, especially in the non-professional journals, of the opposite methods of treatment which have been pursued in Epidemic Cholera; but the contrariety is more apparent than real. The ends in view have generally been the same, but the means adopted have been various, and the disease has often been cured by such as seemed to be opposite in their effects. Thus, different cases have yielded to blood-letting, emetics, opium, or external stimulants, separately employed; while others have been successfully treated by the whole combined.

The desideratum in the treatment, is a medicine, which, by a direct operation, would restore the nervous system to the state which preceded the action of the poison; but it would be eutopian to expect such an antidote. Meantime we can only seek to correct its effects. In contemplating these by the light of the symptoms and appearances after death, there is room for some diversity of opinion on the indications to be fulfilled; and no *methodus medendi*, can, perhaps, be laid down at the present time, against which plausible exceptions in theory, and still more formidable objections in practice, might not be raised.

In malignant cases of Epidemic Cholera, the great indications to be fulfilled are—1. To re-excite the activity of the nervous system. 2. To equalize the circulation, and remove internal congestions. 3. To restore the secretions that are suspended, and improve the condition of those which are perverted. 4. To allay spasms. 5. To moderate the subsequent inflammation and fever. In different cases, different indicacations predominate, and hence the variety in the treatment, whenever it is not purely empyrical. In proceeding to say something of the means of fulfilling these indications, we are not very clear as to the best method to be pursued. If any one of them be accomplished, the malady is vanquished. Thus if the circulation be equalized, the secretions will be restored; and, if the function of innervation can be repaired, the circulation and secretions will improve, and the spasms abate. Several therapeutic agents respectively accomplish two or more of these objects, at the same time; and hence it may, perhaps, be best to treat of them under separate heads.

1. Bloodletting, Cupping and Leeching.—To bleed a patient who cannot be raised from his pillow without fainting, whose pulse is nearly imperceptible, whose skin is cold, and extremities shrunk up to half their ordinary size, would at first view, seem rash and unwarrantable. But experience, which in medicine can grant warrants for any procedure, has sanctioned the use of the lancet even when all these and other symptoms of extreme prostration, are present. To be effectual, or even safe, the blood should be drawn, if posslble, in the first hour of the disease. The quantity to be taken must vary with the effect. It generally flows with difficulty, often *guttatim*, and sometimes not at all, though large veins be opened. In every desperate case, recourse should be had to the jugulars, from which blood will flow when it cannot be elicited from the arms; and, flowing, must contribute more to the relief of the oppressed brain, than when drawn from the extremities. When the loss of a few ounces is productive of still greater debility, the malignity and danger are extreme, and the operation must be terminated. While, conversely, if the blood flow readily, the pulse rise, and no tendency to fainting manifest itself, the prospect is auspicious, and a liberal quantity should be taken. In this case, the powers of the system are less deeply smitten, and the hope of recovery proportionally greater. Of course, blood is not drawn in this stage of the disease and under the symptoms just described, for the cure of inflammation, for none as yet exists; but to diminish the plethora of the great organs, especially the brain, spinal marrow, lungs and heart. That the loss of eight or ten ounces should contribute, perceptibly to this effect, seems rather incredible; but the evidence is conclusive. The number of bloodlettings must be decided by the circumstances of each case. The stage of prostration is generally too brief to admit of many repetitions, and in most cases, a single venesection suffices. The following are the remarks of Dr. Lefevre in reference to this remedy. They were made at St. Petersburgh. We extract from the M. C. Rev.

Bleeding from the arm in the first stage, when the pulse is full and the temperature not reduced, is often sufficient to cut short the disease. The quantity of blood to be drawn should be but small; eight ounces will be sufficient to allow the remainder to circulate more freely and relieve the heart, and this will not too much exhaust the patient.

The blood is generally thicker than usual, highly carbonized, and forms a loose coagulum. I do not know if the blood of Cholera patients has been analyzed during the present epidemic. The patient usually feels immediate relief, particularly where the head has been much affected. He should be bled in the horizontal posture, and remain quiet for some time afterwards. The operation of medicines is generally much facilitated by a small bleeding.

The absence of the pulse is no prohibition to the use of the lancet, unless this is accompanied by other symptoms of great debility, and the system has been exhausted by previous evacuations, and the surface is covered with a cold clammy sweat; in such instances I have never seen blood-letting serviceable, though many assert the contrary. In some cases the pulse ceases to beat very early, but upon opening a vein the blood flows slowly at first, gradually the current becomes fuller and stronger, the pulse beats very sensibly, and the heart thus relieved is enabled to continue the circulation.

To these we shall add from the appendix to Mr. Searle's book, the experience of Mr. Orton and Mr. Chapman. The former remarks:—

I am extremely happy to have it in my power to bear testimony in the strongest terms to the efficacy of the bleeding system of treatment

In four cases I have seen it fail; but in them all, the more severe symptoms had continued from 5 to 13 hours before admission. In 32 others, I have seen it followed by rapid cures, and in 15 of these the second stage had commenced. In none have I seen it fail, when applied before, or soon after the commencement of that stage. One of the above cases was a native, and it was one of the most strongly marked in favor of the practice. The same system has always been pursued to some extent in the natives by Mr. Owen, garrison surgeon here, and with great success.

Mr. Chapman says:—

All the reports which have hitherto been published upon the treatment of cholera, exemplify very strongly the utility of bleeding in the earliest stage of the disease: but it is also observable, that this remedy has been had recourse to in all, even its most opposite stages. In reading the Bombay Report (excellent as it is, in illustrating the good effect of bleeding in the first stage) especially does this become apparent; patients, in the indiscriminate use of this practice, are bled both at a time when the pulse is scarcely operated on by the disease, and also in the latter stage of the malady; when, from the almost total absence of the circulation, blood scarcely can be drawn. The effect of bleeding in the first instance, does in almost every case appear to be marked with a most decidedly beneficial result; and even where recovery has not been the consequence, a retardation of the last symptoms of the disease has certainly been obtained, but whilst on the one hand in the first stage of spasmodic cholera, bleeding appears to be thus beneficial; it is no less evident, that the use of this remedy in the latter stage, is almost uniformly attended with that result, than which no other could be expected. The limited opportunity I have had, in comparison with many others, of putting the practice of bleeding into execution, certainly does not warrant, in itself, the above observation; it has however been the result of the little experience I have had; it is to be observed by the perusal of all the reports upon the subject, and is an inference fairly to be deduced from every analogy which bears upon it.

Cupping and leeching would be adapted to the stage of prostration, if the skin were not so exanguineous, that little blood can be made to flow. Dry cupping, however, might do good. In sporadic cholera, we have repeatedly witnessed its beneficial effects; and our friend, Dr. Heermann, senior surgeon of the navy, has informed us, that in the edemic cholera of New Orleans, he found dry cupping of great advantage.

To the stage of prostration, succeeds, in many cases, the stage of inflammation. When this supervenes, the lancet is employed as an antiphogistic, and must be used in proportion to the intensity of the symptoms.

2. Emetics.—Many of the worst cases of Epidemic Cholera, are not attended with vomiting. That symptom, indeed, seems on the whole to be less violent, than we are accustomed to see it in the endemic of this country. We need not be surprized, then, to learn, that emetics have been administered both in Asia and Europe, with decided advantage. To be efficacious, they should be exhibited early, and the more stimulating should be preferred. If tartar emetic should be chosen, it ought to be dissolved in a fluid that will excite; such as brandy and water, or an infusion of peppermint, cayenne pepper, ginger, or mustard; or paregoric or laudanum should be added to its aqueous solution. As a stimulating emetic, a mixture of powdered mustard in warm water, or a strong solution of common salt, has been found highly efficacious; and is strongly recommended by Mr. Searle. The great power of full vomiting, and this in every case where emetic medicines are used should be the object in view, in reviving the sensibilities of the nervous system, giving impulse to visceral circulation, determining the blood to the surface, and restoring perspiration, have long been known to practical men, and fully explains its beneficial operation in Cholera Epidemica. Vomits are adapted, however, but to the stage of depression. When inflammation and febrile reaction have come on, and the patient labors under gastro-ente ritis, arachnitis, or spinitis, they would of course be inappropriate and often injurious.

The experience of many British practitioners in India, has been decidedly in favor of the emetic practice. We shall lay before our readers a few extracts from Mr. Searle's book:

Mr. Wilson observes:—

> The natives of this country, among whom the disease is by no means unknown, are in the habit of giving to those affected with it, a strong solution of common salt in water; some give the following mixture, one pice weight of onion juice, and two pice weight of arrack; both these have violently emetic effects, but they say they are very successful in treating it in that manner.

The testimony of Mr. Neilson is in part, as follows:

I had been informed that emetics had been used in the spasmodic cholera with advantage, and I gave them a trial, the result of which was very satisfactory, the solution of tartris antimonii, two grains in an ounce of water, was given in half ounce doses every half hour, till free vomiting was produced, and it was encouraged by copious draughts of warm water. The whitish watery matter was at first ejected from the stomach, but the operation of the emetic being promoted, some bile was brought off; and the stools, which had continued whitish and watery, gradually assumed a bilious, or rather, a more natural appearance and a better consistence. As a strong determination takes place to the surface from the operation of the emetic, it thereby produced very beneficial effects; in the body, and particularly the extremities, which had felt cold and chilly, the circulation and natural heat were soon restored; and the pulse, which had been very feeble, or scarcely perceptible, became regular and distinct.

Mr. Barton, who prescribed tartar emetic in large doses, reports its effects in these words:—

At an early and severe period of the prevalence of the disease, when neither the number of cases nor the severity of the symptoms indicated a milder form, the tartrite of antimony was had recourse to; I must confess it was with diffidence, and some degree of anxiety and fear I had recourse to a medicine known to act as a powerful emetic, and in a disease where constant vomiting is so prominent a symptom, accordingly, I used it in the first two or three instances, in doses of one grain with five grains of calomel; its effects emboldened me, and I had it given in doses of two or three grains. One of the first effects of the tartrite of antimony, and what I look on as an important one, was, in almost all cases, bringing off the ingesta, which had been retained, though the vomiting of congee like water had been previously urgent and frequent; ten or fifteen minutes after the ingesta were thrown up, after waiting to see if the stomach was emptied of them, a second dose was administered. Every means of keeping the patients well covered now were strictly adhered to, to cause the tartrite of antimony to act on the skin. The dose was repeated every hour, or two hours, according to the urgency of the case. When the patients could be kept steadily covered, the first favorable symptoms were, a slight degree of heat about the shoulders, which gradually and slowly extended downwards; the countenance lost its collapsed and ghastly look, and the pulse began to be indistinctly felt, and the cold clammy sweats wore off.

Our limits preclude further extracts; but enough has been offered to secure for emetics a favorable estimate, among all who are not governed by prejudice against this class of remedies.

(*Concluded under the Analytical head.*)

### Epidemic Cholera.—An Eclectic Review.

*Concluded from page* 616.

3. Calomel and Purgatives.—As indulgence in eating, is often the exciting cause of Cholera, the stomach and bowels at the onset of the disease, are generally surcharged with recremental matters. Experience has demonstrated, that spontaneons vomiting and diarrhœa do not necessarily rid the canal of these ingesta. Barley water dejections, may continue for hours, and the mucous membrane still remain oppressed and irritated, by half digested aliment. On the early removal of such matters from the stomach, much of the efficacy of emetics manifestly depends. It is equally important to expel them from the bowels; and whenever the alvine evacuations have not been copious and fæculent, cathartics are required. The greatest difficulty in their use, arises from the irritable state of the stomach. That which irritates least, and at the same time is most difficult to expel, is calomel. It may be slow, however, in passing the pylorus, for the action of the stomach is far from favoring its advancement. Should it reach the duodenum, its effects must generally prove beneficial. All the world know, that few remedial agents act so powerfully on the portal viscera; and few things, in clinical medicine, are better ascertained, than the efficacy of this medicine in sporadic and endemic cholera. In the terrible malady now under review, it has disappointed no reasonable expectations. Mr. Searle is its eulogist, and his citations of the experience of others, speak greatly in its favour. It has been given in every variety of dose, from one to twenty or thirty grains, every two or three hours. For ourselves, we have generally seen large portions, at distant intervals, more efficient, in quieting the stomach, and correcting the morbid secretions, than small doses frequently administered. It should be given in powder instead of pills, which are more easily thrown up. Camphor or aromatics, sometimes reconcile the stomach to its impress and are peculiarly proper when the prostration is great. But the most useful adjuvant is opium, of which we shall presently speak.

Other cathartics, than calomel, have been employed, of which the chief are Epsom salt, ol. ricini, and magnesia, the last, especially when the excretions give evidence of acidity. Small and repeated doses of these medicines, seem to succeed better than large portions, Mr. Searle has collected from the practitioners of India, or rather from their Bengal and Madrass reports, a considerable mass of testimony, in favour of this class of medicines, and is lead by its perusal to offer the following remarks:—

Purgatives—this section renders it very clear, that these remedies have not been duly appreciated; on the contrary, the aqueous purging which has been so generally present, and so frequently the first symptom complained of, having led to the inference, that the extreme languor, and depression of the powers of life were in no inconsiderable degree dependant on this symptom as a cause—a primary object of practice arising out of this view, has been, to arrest this evacuation, and to suppress the action of the bowels, as well as of the stomach: but the error of this system I think I have already rendered apparent, if not, the testimony here adduced bears ample proof in favor of the opposite practice; and exhibits purgatives in the character of a most valuable auxiliary, indeed it would be astonishing, that we should have neglected a class of remedies so strongly recommended to us, by the success attending the exhibition of castor oil, by Mr. Duffin, in the same disease of 1787, and the testimony of Mr. Anderson, and other of the Practitioners of those days, in favor of bleeding, purging, and emetics; but for the fact, that our minds were all pre-engaged by the opium and stimulant plan, which was promulgated by authority, on the recommendation of Mr. Corbyn, with whom it was said to have proved so eminently successful.

We are not prepared to sanction copious and repeated purging. Among other sinister effects, it prevents perspiration. In the forming stage of the disease, it might be highly beneficial; in more advanced stages if carried to much extent it could scarcely fail to increase the exhaustion.

In many cases enemata are valuable. When a deficiency of fæculent evacuation, affords ground for suspecting accumulations in the great intestines, although watery discharges are perpetually occurring injections should never be omitted.

4. Antacids. Dr. Granville of London (not experienced in Epidemic Cholera) has published, on that malady, a Catechism for the use of the people. He speaks with great confidence of the virtues of an aromatic alkaline solution, the composition of which he keeps a secret. From the dose in which he directs it, we may conjecture, that it is a solution of caustic potash, united into a liquid soap, with some kind of essential oil. However this may be, we should expect much in certain cases, from such a compound. Magnesia administered in new milk, by Mr. Scott of India, proved successful in some cases, even when the primæ viæ seemed free from acid. Mr. Searle supposes the benefit may be attributable to the milk. The effects observed by Mr. Scott certainly merit consideration.

5. Opium. From the time when Sydenham first poured out his "liquid laudanum," to the present hour, the preparations of opium have been stereotyped prescriptions for cholera. This certainly speaks much in their favour, as few medicines continue long to enjoy an *undeserved* reputation. In the choleras of America, no physician is ignorant of the excellent virtues of opium. Nearly 30 years ago, Dr. Edward Miller, of New York, recommended a combination of calomel and *opium* in the cholera of children; and for most of that time, we have been accustomed to employ it, with satisfactory success.

It appears, however, from the concurrent experience of a great number of practitioners, that the preparations of opium, have not often succeeded in Epidemic Cholera. Both laudanum and solid opium, have been administered in every variety of dose, but the results *on the whole*, have been unfavorable. The chief cause of failure, appears to be the engorged state of the brain—always augmented by narcotics; and keeping this in his eye, the physician will have little difficulty, in deciding on the cases which may or may not, admit of the use of this medicine. In full habits, its effects must of course be prejudicial, and, in most cases, it must follow, not precede venesection. Every experienced physician, knows that blood-letting is the proper predisposing measure for the use of opium. With due reference to the condition of the brain and bowels, this narcotic may undoubtedly be given with much comfort and advantage to the patient. Its power over spasmodic action, when not sustained by continued local irritation or cerebral congestion, is universally admitted; while its *indiscriminate* employment in Epidemic Cholera, or any other disease, must necessarily be attended with sinister effects.

We do not discover, that the salts of morphia have as yet received a fair trial. They might be found more beneficial than solid opium; as morphia is said to affect the brain less unfavorably, than narcotin.

We have so often witnessed the good effects of uniting opium with calomel, in the endemic cholera of this country, that we should be strongly tempted to try that compound, were the epidemic to visit us; always of course, preparing the patient for its exhibition by blood letting and purging.

6. Sudorifics. Epidemic Cholera is attended with such a signal failure of the functions of the skin, that sudorifics are obviously indicated. Some of the British practitioners, in India, have given tartarized antimony for this purpose, with advantage. This practice, at first view reprehensible, may perhaps be worthy of adoption. We should be disposed to recommend the medicine in large doses, combined with opium, in a solid form. In this manner, an antimonial action might be speedily established in the nervous and circulatory systems; which would generally be followed by increased secretion from the mucous membranes and skin. We should feel little apprehension of augmented vomiting from this practice. Ipecacuanha has, also, been employed with advantage, and probably operates as a diaphoretic. But neither of these remedies is adapted to a state of *exhaustion*, however admissible they may be in that of *depression*. We are not aware, that hot water in large draughts has been employed. Might it not prove beneficial? Would it not be absorbed, when the vomiting was not incessant, before its temperature was reduced to that of the body, and by its heat stimulate the heart and other indundated organs into more vigorous action?

7. Diffusible Stimulants. Brandy, camphor, cayenne pepper, the volatile oils of cajuput, peppermint, cloves, cinnamon, and other aromatics, sulphuric ether, and ammoniated alcohol, are among the active stimulants, which have been administered in Epidemic Cholera. The extreme prostration of the vital forces, would seem to call for prompt and powerful stimulation; but although it has often done good, it has still oftener failed, and not unfreqnently proved injurious. When the brain is oppressed, this class of medicines make the matter worse; and in cases less congestive, but more inflammatory, they do harm, by the excitement they create. In persons previously enfeebled, or possessing the lymphatic temperament, they are most likely to prove beneficial; but even to these, they cannot be administered indiscriminately, with advantage; while to those of a sanguine temperament, and vigorous constitution, or whose bowels have ejected nothing but gruel like excretions, and are still loaded with fæculent matters, they cannot fail to do serious injury.

8. Tonics. The analogy between Epidemic Cholera and congestive intermittent fever, has occurred to many physicians, who have witnessed both diseases; and the known efficacy of the sulphate of quina in the latter, has suggested its use in the former. Dr. Hamett (Med. Chir. Rev.) advises its union with calomel. We have fouud the quina to lessen the frequency, and increase the fullness of the pulse, tranquilize the nervous system, and promote perspiration; effects, which, produced in cholera, could not fail to prove beneficial. Engorgements of the viscera, in malignant intermittents, constitute no prohibition to the use of this medicine; which more certainly relieves them than any other; and why then should it not be administered, in cholera attended with the same congestions ? As to other tonics, they would seem to be indicated only, or chiefly, in the stage of convalescence.

9. External Applications. The centripetal tendency of the fluid in Epidemic Cholera, has suggested to the profession and the people, wherever it has prevailed, an early and persevering resort to external applications. When successfully used, they revive the sensibility of the skin; refill it with blood, and restore the lost functions of calorification and perspiration. In proportion as these effects are brought about, the internal organs are relieved of plethora and irritation; the heart and brain, being liberated, act with greater regularity and power; the secretions are revived, and the spasms cease; though certain parts may simultaneously pass into a state of serious inflammation.

Caloric has been the active agent in most of the internal applications which have been used. 1. The hot bath, made stimulating with salt, mustard, or ardent spirits. Although this has been lauded by some, the greater number of physicians have preferred other modes. The time necessary to the preparation of the bath; the absence in most families of a bathing tub, in which the patient can lie down; the danger of his sitting up; and the exhaustion of his strength in getting in and out of the bath, are practical objections of some moment; but it has been thought, that, independent of these, the liquid medium is less beneficial than a gaseous or solid. Hence, 2, hot air and the vapour of burning spirits, have been preferred by many practitioners. For applying the latter, the portable vapor bath of Dr. Jennings might be employed. 3. The patient, or his lower extremities only, may be surrounded with hot ears of Indian corn, or bags of hot meal or ashes. 4. Frictions, with dry and hot flannels, bags of dry sand or salt, rags dipped in spirit of turpentine or tincture of cantharides, or impregnated with powdered mustard; lastly, the warm hands of the attendants, unarmed with any medicinal agent. In most of these methods, the effects depend chiefly on the application of heat, but its efficacy is greatly augmented by friction; and, hence, of all the applications to the skin, those mentioned under the last head, seem to have been most beneficial. 5. Sinapisms, cataplasms with Cayenne pepper, and blisters, applied to the epigastrium, spine, and insides of the thighs and arms, are not to be omitted, except for more speedy and potent modes of stimulation. 6. In this Journal, Dr. Awl, of Ohio, has recommended from experience, when the limbs are cold and shrivelled, in endemic cholera, to cover them with tight flannel rollers, and saturate these with hot oil of turpentine; a powerful application certainly, and one which seems well adapted to the epidemic disease, in which the bandages, by compression, might tend to repress the spasms and cramps of the limbs.

Having, thus, given a general view of the remedies that have been found most efficient in the treatment of Epidemic Cholera, we shall refer to Mr. Searle for detailed instructions, as to their application in ordinary cases, and close this part of our task with the following extract:

The patient, on the first symptom of affection, to be confined to the recumbent posture, in short be lain in bed, between a pair of warm blankets, in an open airy room—when, should there be symptoms of indigestion, or the stomach appear oppressed, it will be advisable to evacuate it, by the patient either drinking plentiful of warm water, or by what is better—a mustard emetic, which operates as a powerful and general stimulant on the system: two tablespoonsfull of the flour of mustard, or the same quantity of the powder, of the common black mustard seed, )which is to be procured in every bazaar in India, and which I found to be decidedly more active than the Europe flour) given in half a pint of pretty warm water, operates effectually in a few minutes, without inducing any subsequent feelings of exhaustion; on the contrary, the eyes sparkle, and a genial glow of warmth succeeds throughout the system, with proportionate increased vigor of the circulation.

After the operation of this, rather preparative measure—to the stomach's being placed in the best condition, to be acted upon by our remedies more particularly curative,—and in furtherance of the same, a warm clyster should be administered, consisting of a dessert spoonful of table-salt dissolved in a pint of warm water, and a spoonful of common, or castor oil; and the same should be repeated every half hour, or oftener—for by thus keeping the bowels excited, tranquility of the stomach is insured, and the consequent retention of our remedies—the chief of which, is calomel; but as this requires some time before it can be received into the system, and effect its operation—as the absorbing power of the stomach, or susceptibility to its influence—in this disease, the distinguishing character of which, being, the subduction of all the powers of life—is greatly diminished, it becomes necessary to give it in proportionately large doses, and at the same time, in aid of its operation, and in support of the living powers—to administer occasional cordials. A scruple of calomel in powder should therefore be placed upon the tongue, and the patient gargling his mouth with a little brandy and water, should swallow it; but it must be observed, the quantity of the latter should at no time exceed three tablespoonsful, which may be in the proportion of one of spirit, to two of hot water,—as a bulk of any fluid, in a delicate irritable state of the stomach, is invariably productive of its rejection.

If the case is urgent, the same dose of calomel may be repeated every hour; otherwise, in two hours; or if the patient is much improved, in half the quantity; and thus prolonging the interval, or reducing the quantity—it must be continued, according to the state of the patient, till bilious stools and urine are produced;—the spirit and water, or mulled wine, either; or where the system is very low, thirty drops of (sal volatile) aromatic spirits of ammonia. or of hartshorn in half a wine-glassful of water, may be singly, or alternately administered, every quarter or half hour; with the precaution before given, to avoid oppressing the stomach by undue quantity.

In addition to these means, if the skin is cold, warm flannels should be constantly applied; or if the skin is damp and the patient suffers by cramps in his legs and arms, the parts may be well compressed, and rubbed with the flannels besprinkled with hot salt. We have yet omitted to mention a very important remedy, one capable of producing much good or no less harm—this is blood-letting—which if the patient is an European, or native of pretty robust habit, should be early resorted to—if the pulse admits of it, that is, if compared with another persons—it is of pretty moderate strength: the object to be bore in mind by bleeding in this case, is to excite, by removing oppression from the brain and circulation, and not to subdue the action of the heart, that it should be taken from the patient whilst continuing in the recumbent posture,—and here I must insist once for all, that on no account and for no purpose is the patient to be permitted to sit up, or leave the recumbent state, or sickness almost immediately takes place; the evacuations should therefore be received in a bed-pan or cloth; or the blood be taken from a rather small orifice, that, the stream being in consequence small, the system may have time to accommodate itself to the deprivation,—the effect of which, however, should be carefully watched—the operator keeping his finger during the time on the pulse, at the same time encouraging the patient by suitable conversation;—when, at the instant it is found to flag, without reference to the quantity withdrawn, whether much or little, the finger should be placed over the orifice; but it must be borne in mind, that fear, nausea, or sickness may occasion this result, that should the quantity taken have been small after a few minutes—if the pulse recovers its wonted strength, as it is an object to carry it to as great an extent as the circumstances of the patient admit—the finger may be removed from the orifice in the vein, and the blood allowed again to flow, with the precautions before specified; but should after a further small loss, the same result ensue, it is clear that any additional attempt at this time, would be injurious; though it may be afterwards practiced, as excitement becomes developed, either in relief of spasms, sense of burning heat in the stomach, or pain in the head, or oppression of breathing: and with the precautions I have given, may be frequently put into practice, and without the possibility of harm—but on the contrary with the happiest effect; for in this disease small bleedings in relief of the engorgement of the brain, stomach, and heart, are clearly and most forcibly indicated. The same intention is partially fulfilled by the clysters, but as warmth and excitement become developed, evinced too by the desire the patient has for cold water—these may be aided, or superceded by a weak and cold solution of Cheltenham, or Epsom salts, or of cream of tartar, with which the patient may be now indulged—in the quantity of a wine-glassful at a time, instead of the cordials—which would now prove injurious; these will not, however, supercede the calomel, the necessity for which still continues, not only till bilious stools are procured, but even then, though in smaller doses, till healthy evacuations follow. It may, however now, on febrile symptoms taking place, be well to combine it, with an equal weight of James's fever, or antimonial powder, and give it, if it is preferred, in the form of pill; but mind if the calomel is thus combined, acids, such as cream of tartar, are not admissible, as an emetic compound would be the result. The calomel and antimonial powder we would now advise, in the proportion of two grains of each, every two hours, with a tea-spoonful of Epsom or Cheltenham salts, in a claret-glassful of water with every second dose, and if there is much thirst, the patient may at the same time be allowed, a wine-glassful of barley or cold water every half hour; and the same be continued, till the secretions of bile and urine are restored—when, and not before, may the patient be allowed some sustenance, the best of which will be *light* beef tea, or chicken broth, for it must be remembered, and borne in mind, during the convalescence, that in proportion to the feeble state of the patient, so is the stomach weak, and powers of digestion.

Many have an objection to salts, where this is the case, two table spoonsful of castor oil may be substituted, or a dose of rheubarb and magnesia when this is preferred. Should the operation of the purgative be attended with much exhaustion, it may be necessary to support the patient with some spiced broth, wine and water, or mulled wine; or it may even be necessary to moderate it if there is much sinking—by a dose, of from twenty to forty drops of laudanum; but this is providing against contingencies, which with moderate care and attention will seldom be found necessary.

The secretions from the bowels are now sometimes so exceedingly acrimonious, that in passing along the line of bowels, and from the anus, they produce, from extreme irritation—very considerable exhaustion; when this is the case it will be advisable, to inject an occasional emollient clyster, of starch or thick conjee water, with oil; to the first of which may be added a tea-spoonful of laudanum, and this repeated if necessary; at the same time hot flannels may be applied to the belly.

In the treatment recommended, we have had in view an ordinary attack of the disease, and coming on with symptoms of indigestion, or stomach derangement; should, however the disease, which it not unfrequently does, have made an insidious approach, under mask of a simple loose state of the bowels, and from the continuance of which, the patient is much exhausted; in a case of this sort it may be necessary to quiet the system, and arrest further action of the bowels, till a certain degree of excitement of the system becomes developed; this may be effected, by adding a grain of opium to the first dose of calomel, or an equal proportion (thirty drops) of laudanum; and which it may be necessary to repeat, but it is only under these and the like circumstances that opium can be recommended. The treatment in other respects becomes the same, save, that in such cases, rhubarb and magnesia, or castor oil is to be preferred to salts, in the stage wherein these are recommended; and that bleeding and clysters can only become necessary, in the relief of the symptoms we have pointed out, when excitement becomes developed: excepting when there is experienced, a sense of oppression and distention about the stomach and bowels, when the clysters may be advisable; otherwise it is to be feared, the stomach will be effected with sickness, which will obviate all our attempts at relief. Should there be burning heat of stomach, while at the same time the body is death-like cold, it will be in vain to attempt resuscitation with stimulants, calomel is the only one admissible,—the stomach being in a state of inflammation, a scruple may be given every hour, and at the same time, as there is a great desire for cold water, and nature's craving should be respected, a table-spoonful or two may be allowed every five or ten minutes, but not more at a time, as the stomach is in this state very irritable, and were a quart to be given, it would not satisfy the patient, who would desire as much more five minutes afterwards; that this precaution is indispensable.—The clysters are also in this case to be continued; and should the sense of heat in the stomach be great, a dozen leeches may be applied over the part, and after their removal a flannel wrung out in warm water may be applied, and with this covering exposed to the air, so as to encourage a little evaporation if gateful to the feelings of the patient; it should however be observed, that the temperature be not too much reduced in this way.—If the pulse admits of it, or as general excitement becomes developed, bleeding may be resorted to, particularly if there are spasms, with the precautions I have already enjoined in having recourse to this remedy.

There is another remedy, simple in the extreme, always available, and speaking both from personal experience and observation, I may add, of powerful operation, which is the fan or hand punkah—it not only renews the air, but condenses it, and thus aiding the respiratory function, assists in no inconsiderable degree in supporting the actions of life: that I cannot too earnestly recommend its uninterrupted use from the earliest period of attack; it is at the same time exceedingly grateful to the patient. pp. 114—121.

IV. Prevention.—The extent to which we have already carried this article, requires that we should treat the present head with brevity. Until the cause of Epidemic Cholera shall be known, our means of prevention must be limited to the avoidance of such things, as are observed to favor its action on the body. Several of these have been noted in Asia and Europe, and are nearly the same, which in all countries, have, from time immemorial, been observed to impart activity to the unseen poisons which generate epidemics.

1. Exposure of the surface of the body to a cool and damp atmosphere, especially that of the night, after the intense heat of the day. We have repeatedly known this to excite cholera infantum.

2. Living and lodging in close, damp and unventilated streets, in the midst of domestic filth.

3. Subsisting on a meagre and insufficient diet; a cause not likely to be operative in the United States, except among the slaves of the South. Long fasting in the morning, allied to the same cause in its effects, should be avoided.

4. Excess in eating. Cholera has repeatedly made its attack immediately after such indulgences, especially when crude, indigestible, ascescent, or irritating articles, have been ingurgitated, in bulk.

5. Intemperance in drinking. It is no assertion of our Temperance Societies, that drunkards have been found peculiarly liable to Epidemic Cholera. The most ample experience in Asia and the north of Europe, has demonstrated this important fact; which should be disseminated far and wide, not because it is likely to deter those who are prone to drunkenness, but because the theory of benevolent operations calls for its publication.

6. An irregular state of the bowels. This should be corrected by dietetic means; or the occasional use of a "blue pill" at bed time, and a little Epsom salt in the morning.

7. A bad state of the skin. This should be avoided by frequent ablutions, by frictions, and the use of thin flannel, or muslin next the surface throughout the summer. Passing a towel or sponge over the whole body, and then rubbing it dry with a coarse towel every morning, would promise much for the same purpose.

8. Apprehension of the disease. When excessive, this state of mind has been observed to predispose to attacks. In Europe it has been epidemic under the playful title of *choleromania*. It has every where preceded the disease itself, as

"Coming events cast their shadows before them."

It is the duty of medical men to labor against this unpropitious panic, by inspiring public confidence in the preventive and curative resources of the profession; which are, in fact, very great, as might be shown, did our limits admit of the necessary statistical views. They may perhaps make a part of our next article.

We have been often asked, will the Epidemic Cholera reach America? To which we make this public, and we hope, satisfactory reply—

"The wind bloweth where it listeth, thou hearest the sound thereof, but cannot tell whence it cometh or whither it goeth."

D.

---

### POSTSCRIPT.

Since closing our review, a work on Cholera, consisting of two reports of the Royal Academy of Medicine, of France, to the Minister of the interior, has reached us. We shall notice it fully in the next number. Meanwhile, we offer our readers the following summary of the symptoms and treatment, which we find near the close of the second report, dated Sept. 13, 1831.

Neither should we confound epidemic cholera with sporadic or indigenous cholera, if we may use the expression. The latter, which is observed almost every where and at the same time with the diseases of summer and autumn, is less acute, less violent and fatal: it never passes beyond a certain extent of country, and attacks but a very small number of persons at the same time.

The description of the symptomatology of the cholera may be recapitulated as follow: physicians will easily recognize it by these characters:

Epigastral pains and anxieties; repeated vomitings, frequent stools; the matters voided consisting at first of recently indigested substances, soon appear fluid, whitish, flocculent; violent cramps in the superior and inferior extremities; coldness of the body and abdomen; suppression of urine; the skin of the extremities, of the feet especially, pale, humid and wrinkled; tongue white, moist and cold; peculiar expression of the countenance; distortion of the features, hippocratic face; respiration scarcely perceptible; sinking and disappearance of the pulse.

Now, as it concerns the treatment, we may say in general, that, in the first period of the disease, which is characterized by coldness of the surface of the body, and concentration of the vitality internally, we would advise frictions, either dry or compound; the radiation of caloric to the surface, by every appropriate means; warm covering; vapour baths, various rubefacients, cuppings, sinapisms and blisters.

It is also with the view of bringing back the circulation to the circumference, in very young persons of a robust constitution, that bleeding has been happily employed at the period of imminence, and as near as possible to the moment of invasion of the disease.

In this period may also be administered internally and with advantage, very warm aromatic infusions; such diffusable stimulants as the irritability of the stomach will permit; aromatic oils combined with alcohol and in conjunction with laudanum, either ammonia, James's powder, Dover's powder, &c.

The special alteration of the gastro intestinal mucous membranes has been combated by calomel, rhubarb, aloes, magnesia, either separately or in combination, giving them according to the indications furnished by the individual constitution.

At the nervous period, with typhoid tendency, and even with mutations or transformations of the cholera into typhus—cinchona, musk, valerian, bismuth, camphor oil of cajeput have been given, together with such other means as are generally adopted in the treatment of typhus.

With the view of attacking the prominent symptoms of the disease separately, there have been given,

For the purpose of arresting the vomitings, Riverious' draught, opium cold drinks, and ice;

Against the frequency of the stools, injections containing landanum into the rectum, aromatic frictions upon the abdomen, and sinapisms;

Against the pains and contractions of the muscles, frictions with the oil of turpentine, or oil of cajeput; and these means have appeared so much the more efficacious, when they concurrently tended to warm and stimulate the chilled surfaces of the skin, and rouse the diminished energy of the nervous function, so remarkable in this disease.

---

### RESIGNATION.

Dr. Drake has resigned the Chair of Clinical Medicine, in the Medical College of Ohio.

